# *Cryptosporidium* infection in young dogs from Germany

**DOI:** 10.1007/s00436-022-07632-2

**Published:** 2022-08-26

**Authors:** Lea-Christina Murnik, Arwid Daugschies, Cora Delling

**Affiliations:** Institute of Parasitology, Center for Infectious Disease, Faculty of Veterinary Medicine, Leipzig University, An den Tierkliniken 35, 04103 Leipzig, Germany

**Keywords:** *Cryptosporidium*, Dog, Germany, Prevalence, Zoonotic disease

## Abstract

*Cryptosporidium* is an enteric protozoan parasite which is able to cause severe gastrointestinal disease and is distributed all over the world. Since information about the prevalence of cryptosporidiosis in German dogs is rare, the aim of this study was to examine the occurrence of *Cryptosporidium* spp. in dogs and the potential zoonotic risk emanating from these infected animals. In total, 349 fecal samples of 171 dogs were collected during the dogs’ first year of life. The samples were examined for *Cryptosporidium* spp. using PCR, targeting the small subunit ribosomal RNA gene (*SSU rRNA*). Further analysis of *Cryptosporidium parvum* and *Cryptosporidium canis* positive samples was accomplished using the 60 kDa glycoproteine gene (*GP60*). Overall, 10.0% (35/349) of the specimens were tested positive for *Cryptosporidium. Cryptosporidium canis* was found in 94.3% (33/35) of these samples and the zoonotic type *C. pavum* in 5.7% (2/35). Both *C. parvum* infections were subtyped as IIaA15G2R1. Sixteen of the *C. canis* positive samples were successfully amplified at the *GP60* gene locus. These isolates were identified to belong to the subtype families XXd, XXe, or XXb; however, 2 samples could not be assigned to any of the described subtype families. Considering the close contact between pets and their owners, dogs may act as a potential source of infection for human cryptosporidiosis. The results of this study, in context with other studies from different countries, provide important further insights into the distribution of *Cryptosporidium* species in dogs and their zoonotic potential.

## Introduction

*Cryptosporidium* is an ubiquitous enteric protozoan parasite that causes gastrointestinal disease in a variety of vertebrate hosts. Transmission occurs via the fecal–oral route due to contaminated water or food or by direct contact with the agent (Ryan et al. 2014). To date, the genus *Cryptosporidium* includes 45 different species (Khan et al. 2018; Ježková et al. 2021). Although infection in dogs has been associated mostly with *Cryptosporidium canis* (Uehlinger et al. 2013; Gil et al. 2017; Itoh et al. 2019; Julien et al. 2019), the zoonotic species *Cryptosporidium parvum* has been detected also (Giangaspero et al. 2006; Sotiriadou et al. 2013; Alves et al. 2018). In addition, other species like *C. muris* and *C. meleagridis* have been found occasionally (Hajdušek et al. 2004; Lupo et al. 2008; Ryan et al. 2014). Cryptosporidiosis typically manifests in acute to severe diarrhea along with abdominal pain. In immunocompetent individuals, the course of infection is normally self-limiting. Dogs rarely show signs of infection and act as asymptomatic carriers (Thompson et al. 2008).

Considering the close contact between companion animals and their owners, the role of dogs as a potential risk factor for the infection with zoonotic pathogens comes to the center of attention (Overgaauw et al. 2020). In Germany, 1190 cases of human cryptosporidiosis have been registered in the year 2020 (Robert-Koch-Institut 2020). Most of the human cases are caused by *C. hominis* and *C. parvum* (Xiao and Feng 2008); nevertheless, the so-termed dog-specific species *C. canis* has been found in immunocompromised and immunocompetent humans as well as in children (Pedraza-Días et al. 2001; Xiao et al. 2007a; The ANOFEL Cryptosporidium National Network 2010).

In consideration of these findings, dogs may act as a source for human infection with cryptosporidia. Only a few studies examining the prevalence of dog cryptosporidiosis in Germany have been reported so far (Cirak and Bauer 2004; Sotiriadou et al. 2013; Gentil et al. 2017; Raue et al. 2017), and little is known regarding the occurring *Cryptosporidium* spp. in German dogs (Sotiriadou et al. 2013). The aim of the present study was to identify the prevalence of *Cryptosporidium* spp. in young dogs from Eastern Germany. In addition, the species were examined to assess the zoonotic potential by molecular methods.

## Material and methods

### Fecal samples

In this study, 171 dogs from Eastern Germany (Saxony, Saxony-Anhalt) were included and sampled three times during their first year of life. The dogs originated mainly from commercial breeders, whereas only few samples originated from dogs living in animal shelters (20/349) or in private households (11/349). In the period from July 2020 to January 2022, 349 fecal samples were collected and analyzed for intestinal parasites. Usually, the first fecal sample of each dog was collected at the age of about 8 weeks by the breeders. Six of the samples were sent in as a pooled litter sample, originating from 5 to 10 puppies. Afterwards, the puppies were sold and the new owners were asked to send in samples of the dogs at an age of approximately 5 months and 9 months. Depending on the age of the dogs at the time of sample submission, the samples were organized into 4 groups according to age: 0–9 weeks old, 10 weeks to 5 months, 6–9 months, and 10–12 months of age. The number of samples in each group was 129, 83, 95, and 41, respectively. Some of the owners missed one or more requested submissions despite repeated reminders for unknown reasons. Therefore, the amount of samples in the different age groups differs from the overall number of participants.

On a questionnaire, the owners were asked to score the consistency of the feces (fecal score): 1, firm; 2, soft; 3, mushy; 4, watery; 5, bloody. The fecal score was modified based on the template “Purina Fecal Scoring System” (Nestlé Purina PetCare Deutschland GmbH, Euskirchen, Germany).

Fecal samples were collected on three consecutive days and shipped to the Institute of Parasitology (Faculty of Veterinary Medicine, Leipzig Germany) for parasitological analysis. The fecal scoring was verified after the arrival of the samples in the Institute. The samples were stored at 4 °C until further processing was conducted within 1–3 days.

### DNA extraction

First, all samples were treated with ultrasonics for 5 min. Afterwards, DNA was extracted from each sample using the QIAamp® Fast DNA Stool Mini Kit (QIAGEN, Hilden, Germany) according to the manufacturer’s instruction. Purified DNA samples were stored at − 20 °C until further analysis was performed.

### PCR amplification of Cryptosporidium spp

#### SSU rRNA gene

The detection of *Cryptosporidium* spp. DNA was conducted using a nested PCR protocol targeting the small subunit ribosomal RNA (*SSU rRNA*) gene as described previously (Xiao et al. 1999). The primers used in the PCR reaction are listed in Table 1.

**Table 1:** Primers used for the PCR

Gene	Primers’ first reaction	PCR product length	Primers’ second reaction	PCR product length
*SSU rRNA*	Forward(F1): 5′-TTCTAGAGCTAATACATGCG-3′ Reverse(R1): 5′-CCCATTTCCTTCGAAACAGGA-3′	1319 bp	Forward (F2): 5′-GGAAGGGTTGTATTTATTAGATAAAG-3′ Reverse (R2): 5′- AAGGAGTAAGGAACAACCTCCA-3′	834 bp
*GP60*	Forward (F1): 5′-ATAGTCTCCGCTGTATTC-3′ Reverse (R1): 5′-GGAAGGAACGATGTATCT-3′	921 bp	Forward(F2): 5′-TCCGCTGTATTCTCAGCC-3′ Reverse (R2): 5′-GCAGAGGAACCAGCATC-3′	887 bp
*GP60- Canis*	Forward (F1): 5′-ATACTCTGGTCTCCCGTTT-3′ Reverse (R1): 5′-GTACTCGGAAGCGGTGTA-3′	750 bp	Forward (F2): 5′-AAGGCGCCTCACTCATT-3′ Reverse (R2): 5′-TCAGTTAGATATCACCCATTAA-3′	700 bp

^*bp*, base pairs^

The reaction mixture for the primary reaction contained 2.5 µl 10 × DreamTaq Buffer (Thermo Scientific™), 0.8 µl NTPs, 0.5 µl forward Primer (25 µM), 0.5 µl reverse Primer (25 µM), 0.1 µl DreamTaq Green DNA Polymerase (Thermo Scientific™), 3 µl of the DNA sample, and DEPC water to a total volume of 25 µl. The conditions for the secondary PCR reaction were identical, except for the use of the second pair of primers. As template 0.5 µl of the first amplification product was used. The PCR reaction was performed in a Biometra TADvanced thermocycler (Biometra GmbH) with initial denaturation at 94 °C for 3 min, followed by 35 cycles of 94 °C for 45 s, 58 °C for 45 s, and 72 °C for 1 min and a final extension of 72 °C for 7 min. The conditions were identical for the first and second reaction.

#### GP60 gene

*Cryptosporidium parvum* positive samples were further analyzed addressing the *GP60* gene (Glaberman et al. 2002; Alves et al. 2003). The used primers are listed in Table 1.

The reaction mixture contained 2.5 µl 10 × DreamTaq Buffer (Thermo Scientific™^)^, 0.8 µl NTPs, 0.5 µl forward Primer (25 µM), 0.5 µl reverse Primer (25 µM), 0.15 µl DreamTaq Green DNA Polymerase (Thermo Scientific™), 2 µl of the DNA sample, and DEPC water to a total volume of 25 µl. The conditions for the secondary PCR reaction were the same but contained the second pair of primers. As template 0.5 µl of the first PCR amplification product was used. Reaction conditions comprised an initial denaturation at 94 °C for 3 min, followed by 35 cycles of 94 °C for 45 s, 50 °C for 45 s, and 72 °C for 1 min with a final extension step at 72 °C for 10 min.

*Cryptosporidium canis* positive samples were subtyped using the primers as described previously (Jiang et al. 2021). The used primers are listed in Table 1.

The reaction mixture contained 2.5 µl 10 × DreamTaq Buffer (Thermo Scientific™^)^, 0.8 µl NTPs, 0.5 µl forward Primer (25 µM), 0.5 µl reverse Primer (25 µM), 0.15 µl DreamTaq Green DNA Polymerase (Thermo Scientific™), 3 µl of the DNA sample, and DEPC water to a total volume of 25 µl. The conditions for the secondary PCR reaction were identical but contained the second pair of primers. As template 1 µl of the first PCR amplification product was used. Reaction conditions comprised an initial denaturation at 94 °C for 5 min, followed by 35 cycles of 94 °C for 45 s, 52 °C for 45 s, and 72 °C for 80 s with a final extension step at 72 °C for 10 min.

For each PCR reaction, a negative and a positive control was included. PCR products were visualized by gel electrophoresis using a 1.5% agarose gel stained with ethidium bromide.

### Sequencing

The PCR products of the secondary reaction were purified by using the PCR Purification Kit (Jena Bioscience, Jena, Germany) according to the manufacturer’s instructions. The purified DNA was stored at − 20 °C until sequencing was conducted. Sequencing was performed in both directions by Microsynth Seqlab (Göttingen, Germany) using the same primers as described above (Table 1). The resulting sequences were evaluated with MEGA version X. Using BioEdit (version 7.2.5), consensus sequences from forward and reverse reads were created and compared to sequences from GenBank® using the Basic Local Alignment Search Tool (BLAST) to identify the species. The herein obtained *C. parvum* sequences were subtyped according to Sulaiman et al. (2005). The phylogenetic relationship among the *C. canis* subtype families obtained in this study as well as recently published *C. canis* sequences (Jiang et al. 2021) was analyzed by forming an alignment with MUSCLE and constructing a maximum likelihood tree using MEGA version X according to Jiang et al. (2021).

### Data analysis

The obtained data was gathered and analyzed using Microsoft Excel Version 16.57 (Microsoft Corporation, Redmond, USA). Statistical analysis was performed using SPSS statistics 27 (IBM, Armond, USA) and significance was assumed when *p* < 0.05. Binary logistic regression was used to test associations between the categorical variables “age groups of dogs” as well as “fecal consistency,” whereby the outcome was the infection status (0, no infection; 1, infection).

## Results

A total of 349 fecal samples were investigated for the presence of *Cryptosporidium* spp. For statistical comparison, samples of puppies up to the age of 9 weeks were defined as “reference.” Overall, 10.0% of the examined samples were positive for *Cryptosporidium* DNA. Prevalence was highest in the age group “10 weeks—5 months” (20.5% positive) and a statistical significance could be determined (*p* = 0.009) in comparison to the reference age group. Dogs older than 5 months did not display prevalence that differed significantly from the reference (*p* > 0.05) (Table 2).

**Table 2:** Prevalence of *Cryptosporidium* spp. in association with the age and fecal consistency

Variable	Age	*Cryptosporidium* infection (%)		Total	Odds ratio (95% confidence intervals)	*p*-value
		Positive	Negative			
Age group	0 to 9 weeks	10 (7.8)	119 (92.2)	129	Reference	
(*n* = 349)	10 weeks to 5 months	17 (20.5)	66 (79.5)	83	3.065 (1.327–7.079)	0.009*
	6 to 9 months	7 (7.3)	89 (92.7)	96	0.936 (0.343–2.555)	0.897
	10 to 12 months	1 (2.4)	40 (97.6)	41	0.298 (0.037–2.397)	0.255
Fecal consistency	1 (firm)	23 (10.7)	191 (89.3)	214	Reference	
(*n* = 349)	2 (soft)	10 (9)	101 (91)	111	0.822 (0.377–1.795)	0.623
	3 (mushy)	2 (9.1)	20 (90.9)	22	0.830 (0.182–3.784)	0.810
	4 (watery)	-	2 (100)	2	0.000	0.999
	5 (bloody)	-	-	-		

*Significant difference compared to reference *p* < 0.05

The majority of fecal samples was scored as firm (*n* = 214), or soft (*n* = 111). Score 1 (firm) was defined as reference. No statistically significant association was found between the infection status and the fecal score (Table 2). Scores 4 and 5 were not considered for statistical comparison due to only few (score 4) or even lacking (score 5) samples to be categorized accordingly.

The sequence analysis confirmed the finding of two different species of *Cryptosporidium*, *C. canis*, and *C. parvum*. The dog-specific species *C. canis* was found in 33 (94.3%) of the *Cryptosporidium* positive samples and was thus clearly dominant as compared to *C. parvum*. The obtained sequences of *C. canis* DNA showed 99–100% similarity with those available for this species in GenBank®, i.e., the sequences with the accession numbers MN696800.1, MN238765.1, and MN238764.1. Five of the *C. canis* sequences obtained in the current study were deposited in GenBank® with the accession numbers OM780299-OM780303.

**Fig. 1:**
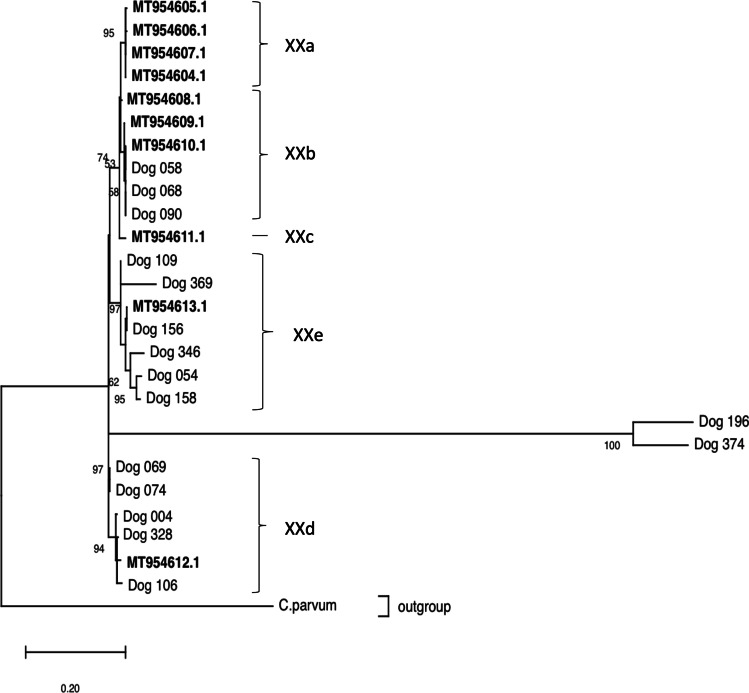
Phylogenetic relationship of the *GP60* gene between *Cryptosporidium canis* subtype families (XXa to XXe) using a maximum likelihood tree. Substitution rates were calculated using the general time reversible model and gamma distribution with invariant sites. The numbers on branches indicate the percent bootstrapping values over 50% by using 1000 replicates. The recently described sequences by Jiang et al. (2021) are in bold letters. A *Cryptosporidium parvum* sequence of the *GP60* gene obtained in this study was used as an outgroup. The assignment to the respective subtype families is indicated with brackets

Furthermore, we were able to amplify 16 of the 33 positive *C. canis* samples at the *GP60* gene locus. Five samples were assigned to the subtype family XXd (accession number MT954612.1), six samples were identified to belong to the subtype family XXe (accession number MT954613.1), three samples were classified as part of the subtype family XXb (accession number MT954608.1, MT954609.1, MT954610.1), and two samples could not be assigned to any of the subtype families (Dog 196, Dog 374). Four of the obtained *C. canis* sequences were deposited in GenBank® with the accession numbers ON820229-32. The phylogenetic relationship among the herein obtained sequences and sequences recently published by Jiang et al. (2021) is shown in Fig. 1.

**Fig. 2:**
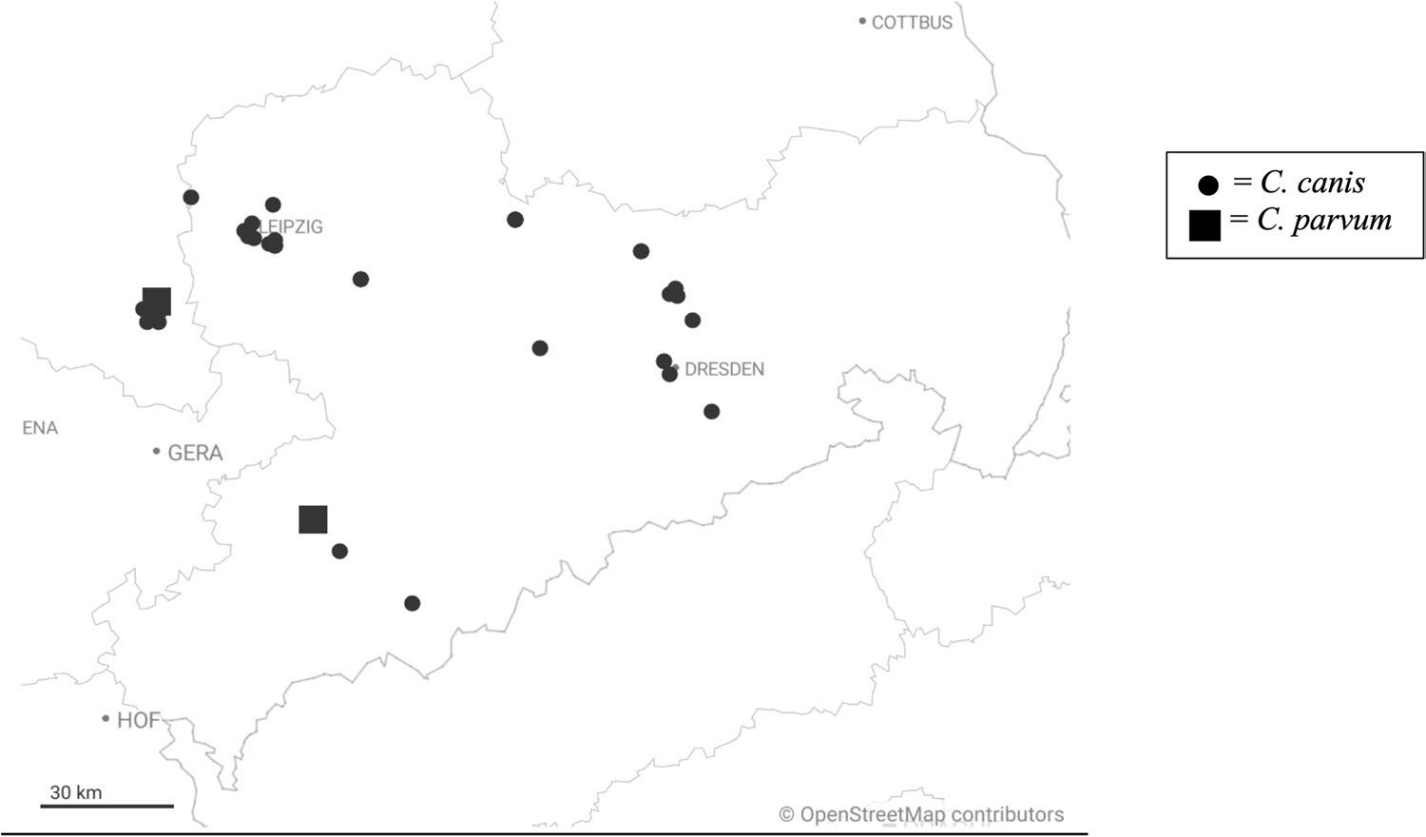
Spatial distribution of samples positive for *C. canis* and *C. parvum* in Saxony and Saxony-Anhalt

Based on the number of TCA and TCG repeats in the 5′ region of the *GP60* gene near the subsequent ACATCA sequence (poly-serine tract) (Sulaiman et al. 2005) found in the herein obtained *C. parvum* sequences, isolates were subtyped as IIaA15G2R1 in both cases (accession numbers OM78517, OM785179). The geographical distribution of all species detected in this study is illustrated in Fig. 2.

Only three dogs (1.8%) were found to be positive for *Cryptosporidium* two times during their first 6 months of life. While in two of these dogs, *C. canis* could be identified at both sampling times, the third dog was infected with *C. parvum* at the first sampling but with *C. canis* thereafter.

## Discussion

The first case of *Cryptosporidium* spp. infection in dogs was described by Tzipori and Campbell (1981); since then, several studies providing prevalence data were published (Abe et al. 2002; Giangaspero et al. 2006; Sotiriadou et al. 2013; Jian et al. 2014).

Worldwide, a broad prevalence range from 0.2 up to 44.1% was described in dogs. The varying prevalence values may reflect variable living conditions (e.g. kennel, breeder or private dogs) (Giangaspero et al. 2006; Hamnes et al. 2007; Palmer et al. 2008; Gil et al. 2017; Sauda et al. 2018; Alves et al. 2018; Itoh et al. 2019; Julien et al. 2019), but different sampling strategies and laboratory methods may contribute to this striking variation. In China and Brazil, prevalence was 6.9% and 10.4%, respectively, irrespective of keeping conditions (Li et al. 2019; Homem et al. 2019) which is quite close to the values found for Eastern Germany in the present study which may indicate that regional differences are not of great impact. In central Italy, strikingly low prevalence of cryptosporidia of 0.2% was reported in 639 kennel dogs (Sauda et al. 2018) whereas values were somewhat higher in an earlier study with 3.3% in 240 kennel and privately owned dogs (Giangaspero et al. 2006). Similar observations were reported from northern Spain, where 4.1% of 194 fecal samples originating from dogs kept in rescue centers were positive (Gil et al. 2017). Another study in northern Spain found 5.5% of 55 privately owned dogs positive for *Cryptosporidium* (de Lucio et al. 2017)*.* In Eastern Spain, 6.8% of 263 fecal samples of dogs within different living conditions were tested positive (Sanchez-Thevenet et al. 2019). In contrast, a much higher proportion of 44.1% of dogs was tested positive for cryptosporidiosis in Norway. In this study, fecal samples of 290 privately owned dogs were probed several times during their first year of life (Hamnes et al. 2007), which obviously explains the much higher prevalence values as compared to studies based on arbitrary single sampling as emphasized by Hamnes et al. (2007).

In our present study, the infection rate with *Cryptosporidium* in young dogs from Eastern Germany was on an average level of 10.0% (35/349). Compared with other studies conducted in other parts of Germany, in which prevalence rates were 1.2% in 81 symptomatic dogs (Sotiriadou et al. 2013), 2.7% in 184 diarrhetic dogs, and 1.2% in 82 healthy dogs (Gentil et al. 2017), the infection rate was distinctly higher in the current study. However, most dogs were asymptomatic. The higher values reported for Germany in our study may be due to the young age of dogs included in our study. The selection of a suitable cohort may considerably influence results of fecal analysis for the respective pathogen. Several studies have shown that young animals are more likely to have a *Cryptosporidium* infection than older ones (Hamnes et al. 2007; Palmer et al. 2008; Itoh et al. 2019; Li et al. 2019; Homem et al. 2019). Hamnes et al. (2007) found a significantly higher prevalence of *Cryptosporidium* in the 3- and 4-month-old groups of individual dogs. These findings conform with the results of the present study, in which dogs of the age group “10 weeks to 5 months” were significantly more often infected with *Cryptosporidium* (*p* = 0.009) than the younger reference group. An explanation for this observation may be the immature immune system of young animals (Pereira et al. 2019). The stress associated with weaning and moving to a new home could have an additional negative impact on the immune system, making young dogs more susceptible to infection.

Raue et al. (2017) reported that only 3.4% of 29 privately owned dogs in Germany were diagnosed positive for cryptosporidia within a period of 10 years. In contrast, Cirak and Bauer (2004) showed a much higher presence of *Cryptosporidium* spp. in 23.0% of 270 shelter dogs in central Germany. Due to the limited number of samples originating from animal shelters, no clear association of keeping conditions and prevalence was obvious in our study. However, it appears probable that cryptosporidiosis is found at higher prevalence rates under unfavorable living conditions of dogs while good health care, sanitation, and keeping dogs as a single pet reduce the risk of transmission and infection. Further studies are needed to evaluate this assumption.

However, apart from frequency of sampling, many other factors may contribute to variable data resulting from prevalence studies in dogs. For instance, the population and the origin of dogs included, the age of the examined dogs, the keeping conditions, general health, and the diagnostic method applied which may considerably influence the prevalence data.

In our study, no significant relation was found between the fecal consistency and the presence of *Cryptosporidium.* This is consistent with the observations of others (Gentil et al. 2017; Itoh et al. 2019; Eze et al. 2019; Li et al. 2019). On the other hand, Taghipour et al. (2020) observed a positive association between a *Cryptosporidium* infection and diarrhea in dogs performing a meta-analysis of published studies. Veterinarians should keep the parasite in mind including a possible zoonotic relevance (discussed below) when presented with a diarrhetic dog, particularly puppies, although the risk of a clinical course of cryptosporidiosis in infected dogs of good general condition is obviously low.

We observed that only three dogs in the cohort studied were infected with *Cryptosporidium* at more than one sampling time point, which is in line with Hamnes et al. (2007). Based on sequence analysis, two of these dogs were infected with *C. canis* at both sampling time points, whereas one dog was first infected with *C. parvum* and then with *C. canis* later on*.* The persistent infection of two dogs with *C. canis* may be due to either chronic infection or re-infection. It appears possible that the immature immune system of the puppies may be insufficient to rapidly eliminate the agent as it can be expected in adult dogs (Pereira et al. 2019). The consecutive infection with two different species in one dog might support the thesis of a species-specific protective immune response, as it has been shown in calves for *C. parvum*, that does not prevent subsequent infection with another species (in this case *C. canis*) (Wyatt 2000). Although immunity to cryptosporidia is assumingly species-specific in general, data are lacking to support this conclusion for *C. parvum* and *C. canis* in dogs.

Altogether, 94.3% (33/35) of the examined dogs positive for *Cryptosporidium* in our study were infected by the “dog type” *C. canis*. Analogous observations were made in studies examining breeding kennel dogs and sled dogs from Japan and Canada, respectively, in which *C. canis* was the only species found (Itoh et al. 2019; Julien et al. 2019). Furthermore, 48% of these samples (16/33) were successfully amplified targeting the *GP60* gene. In contrast to Jiang et al. (2021), who assigned the majority of *C. canis* samples to the subtype family XXa, we found that most samples belonged to subtype family XXe (6/16). Moreover, we were able to assign samples to the subtype families XXd (5/16) and XXb (3/16). Two of our samples could not be appointed to any of the recently reported subtype families (Jiang et al. 2021). One explanation could be the existence of further subfamilies. However, we were not able to amplify the *GP60* gene for all of our *C. canis* positive samples which is in line with Jiang et al. (2021).

After the first report of *C. canis* in a HIV-positive patient by Pieniazek et al. (1999), this parasite was repeatedly detected in immunosuppressed humans, drawing attention to dogs as a possible source of infection for these persons (Lucca et al. 2009; Sannella et al. 2019). Furthermore, Xiao et al. (2007a) detected *C. canis* in two diarrhetic children from Peru. These children lived together with a dog that excreted *C. canis*. Strikingly, *C. canis* was also detected in immunocompetent humans in the UK, as well as in France (Pedraza-Días et al. 2001; The ANOFEL Cryptosporidium National Network 2010). These reports indicate the possibility of transmission of this dog-specific parasite to humans and may be related to a zoonotic risk presented by dogs. Considering this, canine screening for cryptosporidia including differentiation and subtyping of *C. canis* and *C. parvum* appears to be justified, at least in certain scenarios involving, e.g., severely immunodeficient patients or children.

Although *C. canis* is clearly the dominant species of the genus *Cryptosporidium* in dogs, we also found *C. parvum* in two of the examined samples (5.7%)*. C. parvum* is known to have a broad host range including man and is thus of concern as a major zoonotic pathogen. It was previously reported in dogs in several countries. Alves et al. (2018) detected *C. parvum* in 33.3% of the positive samples originating from 128 dogs of various ages, which were under veterinary care in Brazil. In Italy, analogous observations were documented in 240 kennel dogs and privately owned dogs of various ages. Seven out of eight *Cryptosporidium* positive samples were allocated to *C. parvum* which was explained by the authors as possibly being related to livestock farming in this area (Giangaspero et al. 2006). Sotiriadou et al. (2013) detected cryptosporidia in only 1 of 81 samples, and this one sample resembled *C. parvum.* No other studies were published to the best of our knowledge on species allocation of cryptosporidia in dogs in Germany before.

The genotype IIaA15G2R1 of *C. parvum* was identified in our study and was recently reported in dogs in Great Britain for the first time (Rosanowski et al. 2018). IIaA15G2R1 is the most common subtype found in calves and humans in industrial countries (Xiao et al. 2007b; Feng et al. 2013). This is supported by a study from Saxony (Germany), in which IIaA15G2R1 was the most prevalent subtype in calves (Holzhausen et al. 2019). Because of the high prevalence of *C. parvum* in cattle and broad host spectrum, dogs are probably at a higher risk for an infection when living in areas with cattle farms, as proposed by Moreira et al. (2018)*. Cryptosporidium parvum* is an important zoonotic agent causing human cryptosporidiosis (Ryan et al. 2014). An infection dose of as low as 10 oocysts may lead to clinical cryptosporidiosis in humans (Tzipori et al. 2006). Especially immunocompromised humans and children are at risk to develop a severe infection (Bouzid et al. 2013). The role of dogs as a source of *Cryptosporidium* infection in humans has been controversially discussed for many years. As described above, identification of *C. canis* in humans may hint at the possibility of transmission between pets and their owners. On the other hand, in a study conducted in Germany which assessed the occurrence of *Giardia duodenalis* in dogs and their owners, no significant evidence of a zoonotic transmission was detected, and therefore, the zoonotic potential was considered to be negligible (Rehbein et al. 2019). Due to the similar transmission pathway of *Cryptosporidium* and *Giardia*, this conclusion may also be inferred regarding *Cryptosporidium* species. Additionally, in a similar study which was conducted in Spain, a transmission involving *Cryptosporidium* spp. between dogs and humans could not be proven (de Lucio et al. 2017). Therefore, the zoonotic risk presented by dogs in Germany seems to be low, but, nonetheless, should not be ignored considering the high prevalence of *C. canis*. Further research is necessary to evaluate, whether *C. canis* may present a zoonotic risk especially in persons with a deficient immune system and children.

## Data Availability

The material obtained in this study is stored at the Institute of Parasitology, Faculty of Veterinary Medicine, Leipzig University. Representative nucleotide sequences obtained in this study were submitted to the GenBank® under the accession numbers OM780299-OM780303, ON820229-32, OM78517, and OM785179.
